# Cost-effectiveness analysis of nab-paclitaxel with or without relacorilant for platinum-resistant ovarian cancer

**DOI:** 10.1186/s13048-026-02109-7

**Published:** 2026-04-23

**Authors:** Shubin Lai, Shufei Lai, Xiaoxin Xu, Wenhui Huang, Qiuhong Chen, Suling He, Honglin Xue

**Affiliations:** 1 Department of Pharmacy, The 909th Hospital, School of Medicine, Xiamen University, Zhangzhou, 363000 China; 2 School of Pharmacy, Fujian Medical University, Fuzhou, 350005 China

**Keywords:** Platinum-resistant ovarian cancer, Relacorilant, Nab-paclitaxel, Partitioned survival model, Cost-effectiveness

## Abstract

**Background:**

Platinum-resistant ovarian cancer (PROC) has a poor prognosis and limited treatments. Relacorilant plus nab-paclitaxel (RnP) shows clinical efficacy in Phase III trials, but its cost-effectiveness has not yet been evaluated. This study evaluated RnP versus nab-paclitaxel (nP) for PROC from the U.S. payers’ perspective.

**Methods:**

A partitioned survival model (1-month cycle, 5-year time horizon) was utilized to assess the cost-effectiveness of the RnP regimen for PROC, and appropriate parametric functions were applied to fit survival curves and extrapolate 5 years of treatment. Critical clinical data were derived from the ROSELLA trial. Costs and utility values were obtained from U.S. public websites and published literature. The primary outcomes were total cost, quality-adjusted life-years (QALYs), and the incremental cost-effectiveness ratio (ICER), which was benchmarked against a willingness-to-pay (WTP) threshold of $150,000/QALY. A comprehensive appraisal of model robustness was conducted, encompassing both one-way sensitivity analysis and probabilistic sensitivity analyses (PSA), in addition to scenario analyses to probe the conditions for the regimen’s economic feasibility.

**Results:**

Compared with the nP regimen, the RnP regimen incurred an additional treatment cost of $43,161 but gained an extra 0.27 QALYs in health benefits. Ultimately, the ICER was calculated to be $161,753/QALY, which exceeded the WTP threshold of $150,000/QALY. One-way sensitivity analysis demonstrated that the price of relacorilant had the greatest influence on results, with the PSA showing a 38.9% probability of the RnP regimen being cost-effective. The probabilities that the RnP regimen was cost-effective for PROC were 2.3%, 38.9%, and 82.4% at WTP thresholds of $100,000, $150,000, and $200,000/QALY, respectively. Scenario analyses identified a definitive price threshold for relacorilant ($2.262/mg), representing a 7.8% reduction from the current estimated cost, at which the RnP regimen achieves cost-effectiveness. Subgroup analyses found that the RnP regimen was associated with a relative cost-effective outcome in several subgroups: those aged > 65 years, patients with a primary platinum-free treatment interval ≤ 6 months, and patients with a taxane-free interval ≤ 6 months.

**Conclusion:**

From the U.S. payers’ perspective, the RnP regimen is not cost-effective compared with the nP regimen for patients with PROC. Setting relacorilant’s price at $2.262/mg could render the RnP regimen cost-effective.

**Supplementary Information:**

The online version contains supplementary material available at 10.1186/s13048-026-02109-7.

## Introduction

Ovarian cancer (OC) ranks as the most lethal malignancy among gynecological cancers in the United States (U.S.). According to 2025 statistics released by the American Cancer Society and the Surveillance, Epidemiology, and End Results Program, an estimated 20,890 new OC cases and 12,730 OC-related deaths will occur in the U.S. in 2025 [1]. This accounts for approximately 1.0% of all new cancer cases and 2.1% of all cancer deaths in the country [1]. Despite the continuous decline in the incidence of OC attributed to the widespread use of oral contraceptives and the reduced utilization of hormone replacement therapy, over 80% of patients are diagnosed at an advanced Stage Ⅲ/Ⅳ, with a 5-year survival rate of only 51.6% [2, 3].

The standard frontline treatment strategy for advanced OC consists of cytoreductive surgery combined with neoadjuvant or adjuvant chemotherapy, which typically includes platinum-based agents and paclitaxel [3]. Despite the relatively high initial response rates to this regimen, the vast majority of patients eventually experience disease recurrence, with platinum sensitivity being a critical determinant of treatment outcomes. Approximately 20%-25% of patients exhibit primary platinum resistance to initial chemotherapy, while a large proportion of initially responsive patients subsequently develop secondary platinum resistance [4].

Platinum resistance constitutes a key therapeutic bottleneck in OC treatment and is associated with an extremely poor prognosis. For patients with platinum-resistant ovarian cancer (PROC), the objective response rate (ORR) to single-agent non-platinum chemotherapy is only 10%-20%, with a median progression-free survival (mPFS) of 3–4 months and a median overall survival (mOS) of approximately 12–13 months [5]. Even combination therapy with the anti-angiogenic agent bevacizumab only extends mOS to 16.6 months, leading to a modest improvement in efficacy but carrying inherent toxicity risks [4]. It is noteworthy that the immune-based combination of bevacizumab and atezolizumab failed to significantly improve survival, further confirming the poor prognosis associated with PROC [6]. These findings highlight the urgent need for novel, effective, and well-tolerated therapeutic strategies for the treatment of PROC.

Nanoparticle albumin-bound paclitaxel (nab-paclitaxel, nP) offers a superior safety profile over solvent-based paclitaxel by eliminating the need for premedication, alongside enhanced efficacy in PROC [7]. However, the clinical benefit of nP monotherapy is limited, yielding an ORR of 15%-25% and a mPFS of 3.5–4.5 months in this population [7].

The glucocorticoid receptor (GR) can influence the chemosensitivity of tumor cells by regulating the cell cycle and apoptosis-related pathways. As a highly selective GR antagonist, relacorilant competitively inhibits GR activation and blocks the glucocorticoid-mediated protumorigenic effects [8–10]. Preclinical studies have confirmed its potent synergistic antitumor activity with taxane-based chemotherapeutic agents, which may be associated with the drug’s inhibition of GR signaling and enhanced sensitivity of cancer cells to taxane-induced apoptosis [11, 12]. Recent Phase III clinical trial data demonstrate that the relacorilant plus nP (RnP) regimen significantly improved mPFS (6.54 vs. 5.52 months) and mOS (15.97 vs. 11.50 months) compared with nP monotherapy, while reducing the risk of disease progression by 30% [7]. The regimen was not associated with a significant increase in overall adverse events (AEs) or treatment-related mortality, indicating a favorable tolerability profile and promising therapeutic potential for patients with poor-prognosis OC [7].

While the novel combination therapy improves outcomes for patients with recurrent PROC, concerns have been raised regarding its cost-effectiveness. Conducting a cost-effectiveness analysis is therefore crucial for guiding healthcare resource allocation [13, 14]. Given the innovative nature of this therapy and the scarcity of health economic data on relacorilant, this study aims to estimate and compare the cost-effectiveness of the RnP regimen versus the nP regimen for the treatment of PROC from the U.S. payers’ perspective, thereby providing evidence-based support for the rational pricing of relacorilant.

## Materials and methods

### Participants & treatments

As a modeling study based on published randomized controlled trial data, the theoretical patient cohort in this study was derived from participants in the ROSELLA trial. This cohort comprised adult female patients aged ≥ 18 years with a diagnosis of platinum-resistant epithelial OC or primary peritoneal cancer [7], who met al.l the following inclusion criteria: (1) measurable lesions per the Response Evaluation Criteria in Solid Tumors version 1.1; (2) Eastern Cooperative Oncology Group (ECOG) performance status of 0 or 1; (3) normal organ function; (4) prior receipt of 1 to 3 lines of systemic anticancer therapy, with disease progression or intolerance to the most recent regimen; (5) prior receipt of at least one line of platinum-based therapy; (6) documented platinum-resistant disease (defined as disease progression within 6 months of the last platinum-based treatment); and (7) prior treatment with bevacizumab.

Exclusion criteria were as follows: (1) primary resistance to initial platinum-based regimens; (2) disease progression within 1 month after the last platinum-based therapy; and (3) requirement for long-term systemic corticosteroid therapy.

Patients were randomly assigned in a 1:1 ratio to receive RnP or nP regimen. Specific drug dosages and administration schedules for each group were as follows:

- RnP regimen:
  - Relacorilant: 150 mg, administered orally on the day before, the day of, and the day after each nP administration.
  - nP: 80 mg/m², administered via intravenous infusion on Days 1, 8, and 15 of each 28-day cycle.
- nP regimen:
  - nP: 100 mg/m², administered as monotherapy via intravenous infusion on Days 1, 8, and 15 of each 28-day cycle.

### Model overview

**Fig. 1:**
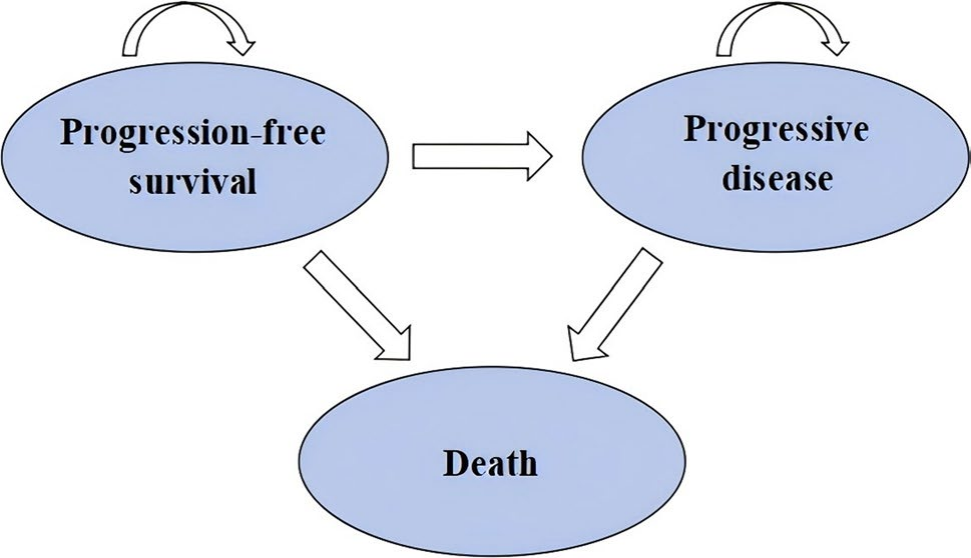
Partitioned survival model states transition diagram

This study adopted the U.S. payers’ perspective to evaluate the cost-effectiveness of the RnP regimen. TreeAge Pro 2022 software was used to construct a partitioned survival model, which incorporated three distinct health states: progression-free survival (PFS), progressive disease (PD), and death (Fig. 1). Initially, all patients began in the PFS state, where they received treatment with the RnP or nP regimen until disease progression, at which point they transitioned to the PD state. Patients in the PD state were followed up every 3 months until death, with best supportive care provided prior to death. The proportions of patients in each health state were estimated using parametric survival functions fitted to the Kaplan-Meier data from the ROSELLA trial.

The model was structured with a 1-month cycle length and a total simulation duration of 5 years. A 5-year time horizon was applied, consistent with mainstream oncology pharmacoeconomic guidelines [15]. Given the extremely poor prognosis of patients with platinum-resistant ovarian cancer, who have a 5-year overall survival rate of 0% [16–18], this time horizon was sufficient to capture all relevant clinical and economic outcomes. In line with standard methodological guidelines for health economic evaluations, a 3% annual discount rate was applied to both costs and health outcomes to account for the time preference of resources and benefits [19]. Primary outcome measures included total healthcare costs, quality-adjusted life-years (QALYs), and the incremental cost-effectiveness ratio (ICER). The willingness-to-pay (WTP) threshold was set at $150,000/QALY, a commonly accepted threshold for assessing the cost-effectiveness of oncology interventions in the U.S. healthcare context [20].

### Efficacy and AEs parameters

Data on therapeutic efficacy and AEs incidence rates for patients were derived from the ROSELLA study [7]. To obtain PFS and overall survival (OS) probability data, the published Kaplan-Meier survival curves from this trial were digitized using GetData Graph Digitizer software. Given the inherent limitation of short follow-up durations in clinical trials, survival data were extended via extrapolation. Specifically, within the RStudio v1.2.5 environment, six parametric distribution models, exponential, gamma, Gompertz, Weibull, log-logistic, and log-normal, were separately applied to fit and extrapolate PFS and OS curves [21, 22]. Model selection was guided by the Akaike Information Criterion (AIC) and Bayesian Information Criterion (BIC), where lower values indicated superior model fit; this quantitative assessment was supplemented with visual inspection of curve alignment. Ultimately, the log-normal distribution provided the optimal fit for PFS and the gamma distribution for OS in the RnP regimen, while the spline distribution was optimal for PFS and the Weibull distribution for OS in the nP regimen. Detailed fitting curves are shown in Supplementary Figure S1. Additionally, the model incorporated grade ≥ 3 AEs occurring at a rate exceeding 3% in any treatment group of the ROSELLA study: fatigue, nausea, anemia, diarrhea, and neutropenia [7]. This threshold is widely used in oncology economic evaluations to focus on clinically meaningful and frequent AEs that substantially affect medical costs and quality of life, while excluding AEs that are rare, require no clinical management, or have negligible impact on cost-effectiveness outcomes.

### Costs and utility values

This study primarily included direct medical costs, covering expenses related to medications, inpatient administration, serious adverse events (SAEs), follow-up costs (such as computed tomography and laboratory tests), and best support care. According to the Centers for Disease Control and Prevention, the estimated average height and weight of adult women in the United States are 161.29 cm and 77.93 kg, respectively; the resulting body surface area of 1.87 m² was used for drug dosage calculation [23]. Assuming that all SAEs occurred only during the first treatment cycle, the expected cost of AEs was estimated by multiplying the unit cost of each AE by its corresponding incidence rate. AE costs were derived from the IBM Micromedex RED BOOK drug pricing database [24]. Due to the unavailability of public pricing for relacorilant, its cost was simulated based on therapeutic equivalence to mifepristone. The equivalence ratio (1,350 mg/cycle relacorilant ≈ 1,800 mg/cycle mifepristone) was derived from clinical trial data demonstrating comparable glucocorticoid receptor inhibitory activity in the target population. Using mifepristone’s verified market price ($552.255/300 mg), the simulated price of relacorilant was calculated as $245.4/100 mg ($2.454/mg) [7, 25–27]. The costs of drugs and additional cost data were obtained from drug information websites and published literature [24, 28–32]. A 3% annual discount rate was applied to both costs and health outcomes [33]. All costs were uniformly adjusted to 2025 U.S. dollar values using the U.S. Consumer Price Index [34].

The health utility values for PFS, PD, and death were set at 0.75, 0.50, and 0, respectively, based on data from previous OC cost-effectiveness studies [22]. Disutility values associated with AEs were incorporated into the analysis to account for their negative impact on quality of life [22, 35, 36]. More details are shown in Table 1.

**Table 1:** Ranges and distribution of parameters used in sensitivity analysis

Variable	Baseline Value	Range		Distribution	References
		Minimum	Maximum		
Costs($)					
Relacorilant per mg	2.454	1.963	2.945	Gamma	^[27]^
Nab-paclitaxel per mg	1.551	1.241	1.861	Gamma	^[28]^
Best supportive care per patient	1484.270	1187.416	1781.124	Gamma	^[31]^
Administration per cycle	230.000	184.000	276.000	Gamma	^[32]^
Cost of follow-up ($)					
Laboratory examinations per unit	300.000	240.000	360.000	Gamma	^[32]^
CT per unit	350.000	280.000	420.000	Gamma	^[32]^
Cost of AEs per event ($)					
Fatigue	313.451	250.761	376.141	Beta	^[24]^
Neutropenia	507.305	405.844	608.766	Beta	^[29]^
Anaemia	5838.488	4670.790	7006.186	Beta	^[24]^
Nausea	3320.813	2656.650	3984.976	Beta	^[24]^
Diarrhea	4580.794	3664.635	5496.953	Beta	^[24]^
Incidence of SAEs in relacorilant plus nab-paclitaxel					
Fatigue	0.09	0.070	0.104	Beta	^[7]^
Neutropenia	0.44	0.352	0.528	Beta	^[7]^
Anaemia	0.18	0.144	0.216	Beta	^[7]^
Nausea	0.04	0.032	0.048	Beta	^[7]^
Diarrhea	0.04	0.032	0.048	Beta	^[7]^
Incidence of SAEs in nab-paclitaxel					
Fatigue	0.02	0.019	0.029	Beta	^[7]^
Neutropenia	0.25	0.200	0.300	Beta	^[7]^
Anaemia	0.08	0.064	0.096	Beta	^[7]^
Nausea	0.03	0.022	0.034	Beta	^[7]^
Diarrhea	0.02	0.016	0.024	Beta	^[7]^
Utility value					
PFS	0.75	0.60	0.90	Beta	^[22]^
PD	0.5	0.40	0.60	Beta	^[22]^
AEs disutility					
Fatigue	0.07	0.056	0.084	Beta	^[35]^
Neutropenia	0.073	0.0584	0.0876	Beta	^[36]^
Anaemia	0.073	0.0584	0.0876	Beta	^[36]^
Nausea	0.07	0.056	0.084	Beta	^[35]^
Diarrhea	0.001	0.0008	0.0012	Beta	^[36]^
BSA (m^2^)	1.87	1.496	2.244	Gamma	^[23]^
Discount rate (%)	3	2.4	3.6	Fixed	^[33]^

*CT* Computed tomography, *AEs* Adverse events, *SAEs* Serious adverse events, *PFS* Progression-free survival, *PD* Progressive disease, *BSA* Body surface area

### Sensitivity analyses

To assess the robustness of the model results, we performed both one-way sensitivity analysis and probabilistic sensitivity analysis (PSA). For one-way sensitivity analysis, the variation range of each parameter was defined based on the 95% confidence interval of the primary data or values derived from relevant literature; in cases where such data were unavailable, the range was set as the baseline value ± 20%. For the PSA, a Monte Carlo simulation with 10,000 iterations was conducted to propagate parameter uncertainty. Most model parameters were assigned specific probability distributions (e.g., gamma distribution for costs and body surface area, beta distribution for probabilities and utility values, and fixed distribution for the discount rate), followed by simultaneous random sampling from these distributions. Additionally, tornado diagrams and cost-effectiveness acceptability curves (CEAC) were generated to visually present the results of the one-way sensitivity analysis and PSA, respectively.

Furthermore, as consensus on the WTP threshold has not yet been reached in the United States, a commonly applied range of $100,000/QALY to $200,000/QALY is typically used. Therefore, we also assessed the probability that the RnP regimen for the treatment of PROC is cost-effective under different WTP thresholds.

### Scenario analyses

Given that the launch price of relacorilant remains unknown, a scenario analysis was conducted to evaluate its economic profile. Specifically, the base-case model was run with varying prices of relacorilant to identify the price threshold at which the RnP regimen for PROC would achieve a ≥ 50% probability of being cost-effective at a WTP threshold of $150,000/QALY.

### Subgroup analysis

A subgroup analysis was conducted to investigate the economic differences in the RnP regimen across patient subgroups characterized by various attributes (such as age, ECOG performance status, prior lines of therapy, prior poly(ADP-ribose) polymerase inhibitor use, and breast cancer gene mutation status). In this analysis, most parameters (such as SAEs incidence and health utility values) retained the settings of the baseline model. Given the data availability, PFS curves for each subgroup were assumed to be consistent with the overall population. Then, the hazard ratio (HR) of OS in subgroups was substituted into the model to calculate the ICERs. The price of relacorilant applied in subgroup analyses was identical to that in the base-case analysis ($2.454/mg, estimated based on therapeutic equivalence to mifepristone), thereby ensuring comparability across analyses.

## Results

### Base-case analysis

Results of the base-case analysis are presented in Table 2. Over a 5-year time horizon, the total cost for the RnP regimen was $58,844, corresponding to a health utility benefit of 1.06 QALYs. In contrast, the nP regimen incurred total costs of $15,682, with a health utility benefit of 0.79 QALYs. The RnP regimen was associated with an incremental cost of $43,161 and an incremental survival of 0.27 QALYs, resulting in an ICER of $161,753/QALY. This ICER value substantially exceeded the commonly cited WTP threshold of $150,000/QALY.

**Table 2:** Summary of cost and outcome results in the base-case analysis

				Probability of Cost-Effective at Different WTP Thresholds($/QALY)		
Regimen	Total cost ($)	QALYs	ICER ($/QALY)	100,000	150,000	200,000
RnP	58,844	1.06		2.3%	38.9%	82.4%
nP	15,682	0.79		97.7%	61.1%	17.6%
Incremental	43,161	0.27	161,753			

*RnP* Relacorilant plus nab-paclitaxel, *nP* Nab-paclitaxel, *QALYs* Quality-adjusted life years, *ICER* Incrementalcost-effectiveness ratio, *WTP* willingness-to-pay

### Sensitivity analysis

**Fig. 2:**
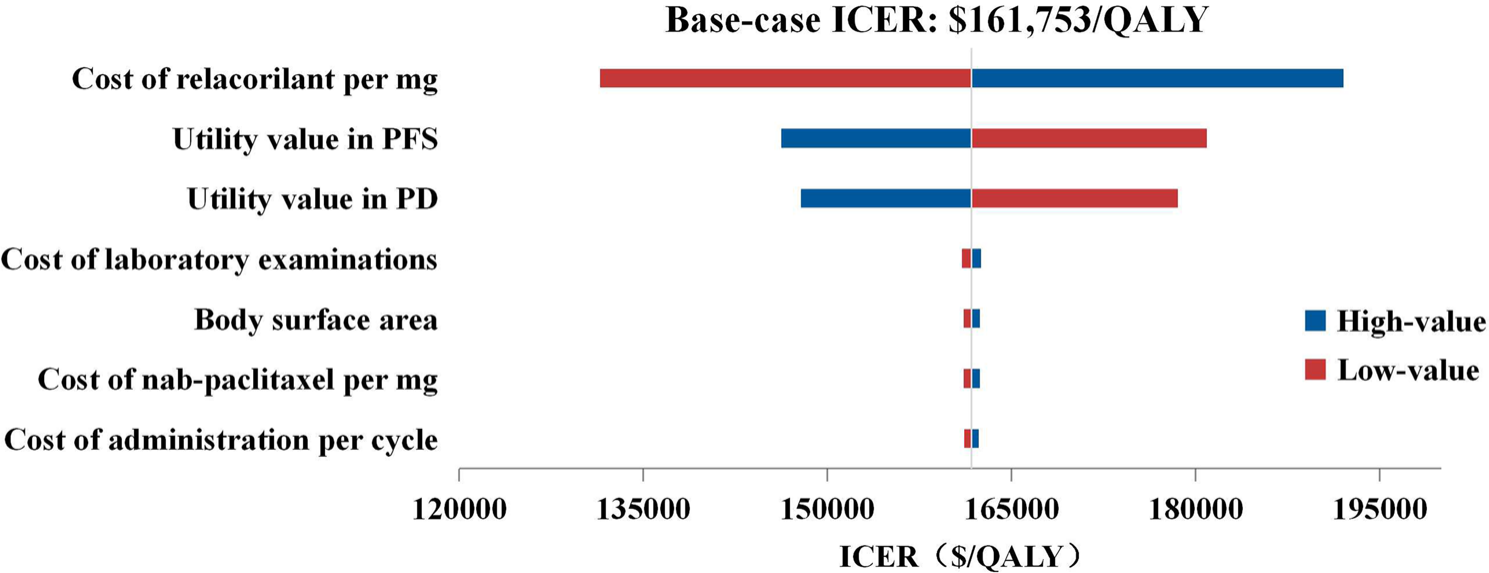
Tornado diagram of one-way sensitivity analysis on the hypothetical price

The results of the one-way sensitivity analysis (Fig. 2) revealed that the ICER was most sensitive to the price of relacorilant and the utility values for PFS and PD. Other parameters, including the cost of laboratory examinations, exerted a limited impact on the ICER. Although adjusting the above three key parameters within reasonable ranges may alter the economic conclusion, the ICER remained above the WTP threshold of $150,000/QALY when most other parameters were varied.

**Fig. 3:**
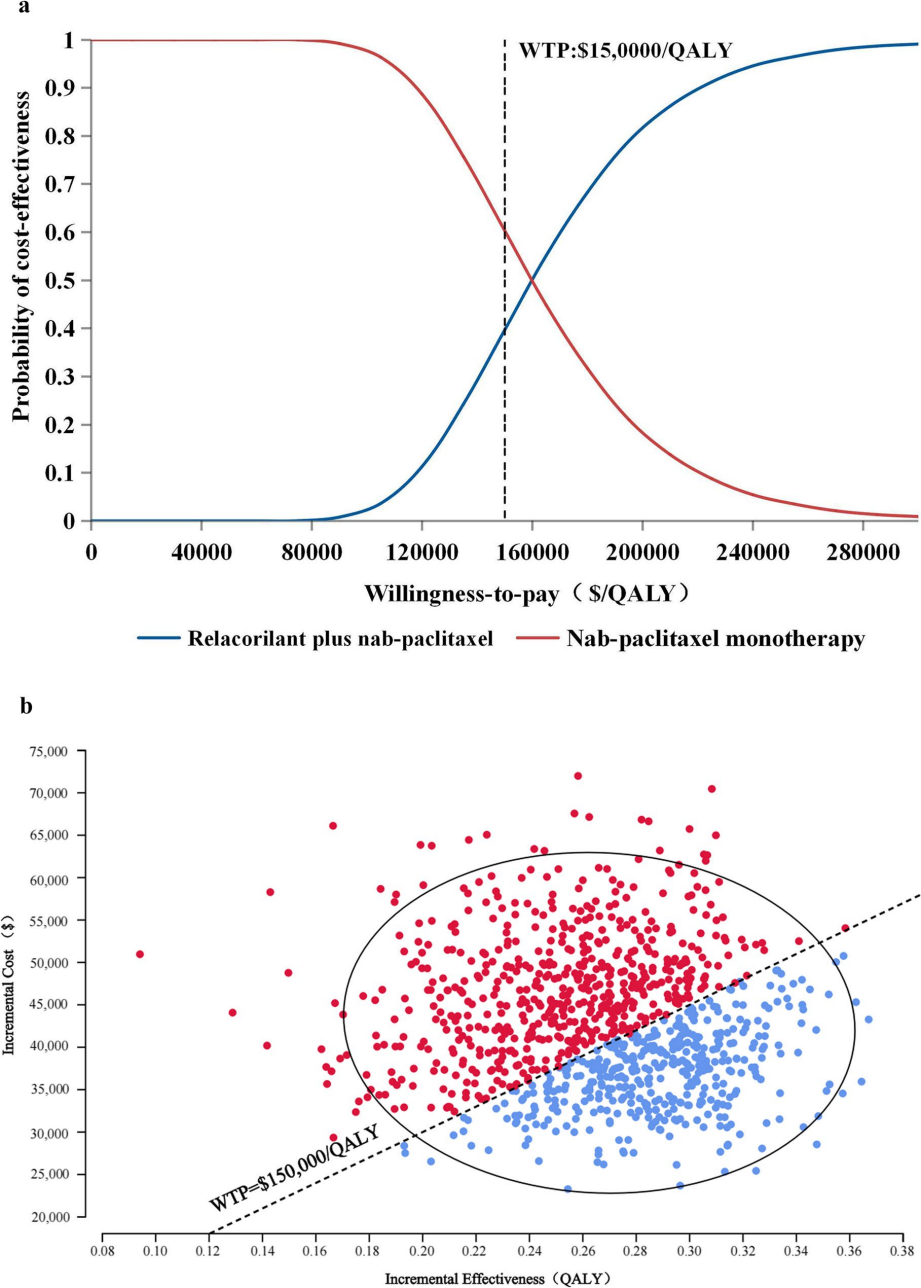
**a**. Cost-effectiveness acceptability curve for probabilistic sensitivity analysis. WTP: Willingness-to-pay; QALY: Quality-adjusted life-year. **b**. Cost-effectiveness scatter plot for probabilistic sensitivity analysis. WTP: Willingness-to-pay; QALY: Quality-adjusted life-year

The cost-utility acceptability curve (Fig. 3a) and scatter plot (Fig. 3b) demonstrated that the RnP regimen had a 38.9% probability of being cost-effective compared with the nP regimen for the treatment of PROC at a WTP threshold of $150,000/QALY. Furthermore, in the U. S., the probabilities that the RnP regimen was cost-effective for PROC were 2.3%, 38.9%, and 82.4% at WTP thresholds of $100,000, $150,000, and $200,000 per QALY, respectively (Table 2).

### Scenario analysis

**Fig. 4:**
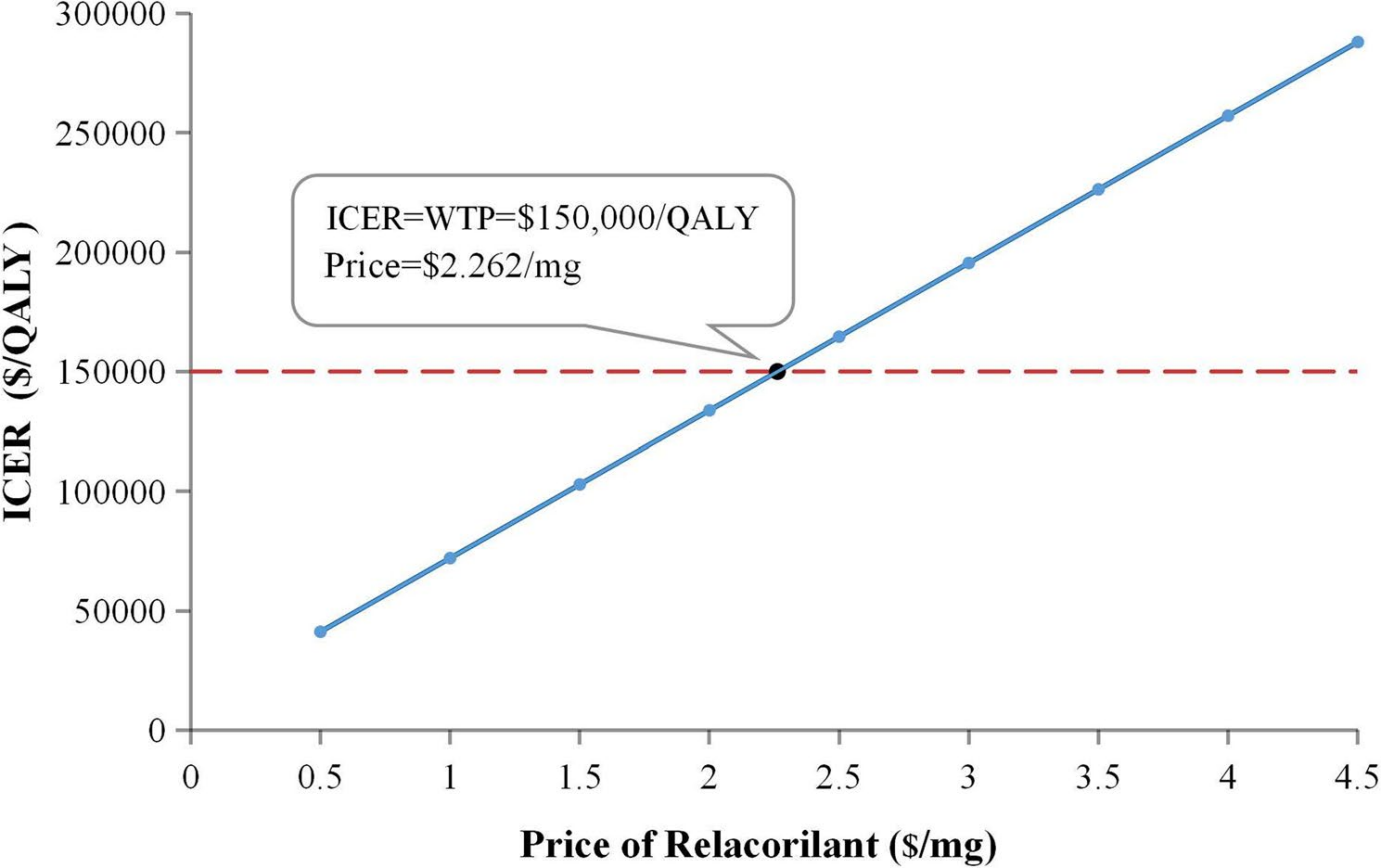
The results of price simulation. The dotted line represents the WTP threshold; the solid line represents the trend line of the ICER scatter point under each price

The results of the price simulation are presented in Fig. 4. Based on the base-case model, the ICER of the RnP regimen for the treatment of PROC reached the WTP threshold of $150,000/QALY when relacorilant was priced at $2.262/mg. Furthermore, PSA demonstrated that the probability of the RnP regimen being cost-effective exceeded 50% at this price point, reaching 51.73%.

### Subgroup analysis

Subgroup analysis results are presented in Table 3. Compared with nP, RnP yielded an ICER exceeding the $150,000/QALY WTP threshold in most subgroups, with a cost-effective probability generally below 50%. Nevertheless, RnP approached cost-effectiveness in several specific subgroups, including patients aged > 65 years, those with a primary platinum-free interval ≤ 6 months, and those with a taxane-free interval ≤ 6 months. The study also found that HR exerted a significant impact on the ICER, indicating that treatment groups with lower HR values exhibited superior clinical efficacy.

**Table 3:** Results of subgroup analysis

Subgroup	OS HR (95% CI )	ICER, median (range) $/QALY	Cost-effectiveness probability of RnP (range)
Age			
≤65 years	0.83 (0.57 to 1.20)	381,269 (159,668 to -2,552,414^a^)	0 (0.407 to 0)
>65 years	0.55 (0.34 to 0.89)	150,341 (79,426 to 494,721)	0.497 (0.990 to 0)
ECOG performance status			
0	0.72 (0.50 to 1.05)	255,595 (129,446 to 1,511,744)	0.01 (0.755 to 0)
1	0.59 (0.36 to 0.97)	169,633 (84,597 to 767,244)	0.313 (0.990 to 0)
Previous lines of therapy			
0	0.80 (0.32 to 1.97)	339,213 (74,503 to -291,764)	0 (0.998 to 0)
1	0.74 (0.49 to 1.12)	273,482 (125,634 to 6,706,496)	0.004 (0.793 to 0)
2	0.66 (0.42 to 1.04)	210,488 (101,775 to 1,354,698)	0.084 (0.931 to 0)
Previous PARP inhibitor			
Yes	0.77 (0.53 to 1.13)	303,779 (141,593 to 12,642,819)	0.003 (0.614 to 0)
No	0.66 (0.42 to 1.05)	210,488 (101,775 to 1,511,744)	0.084 (0.931 to 0)
Primary platinum-free interval, months			
≤6	0.52 (0.31 to 0.89)	137,421 (72,129 to 494,721)	0.668 (1 to 0)
>6	0.82 (0.58 to 1.16)	366,400 (164,568 to -7,895,209^a^)	0 (0.368 to 0)
BRCA1 or BRCA2 mutation			
Positive	0.82 (0.33 to 2.07)	366,400 (76,934 to -197,360^a^)	0 (0.996 to 0)
Negative or unknown	0.70 (0.52 to 0.96)	239,264 (137,421 to 720,171)	0.029 (0.668 to 0)
Taxane in last regimen			
Yes	0.77 (0.40 to 1.49)	303,779 (95,751 to -478,366^a^)	0.003 (0.961 to 0)
No	0.66 (0.48 to 0.92)	210,488 (121,931 to 574,077)	0.084 (0.825 to 0)
Taxane-free interval, months			
≤6	0.57 (0.26 to 1.26)	159,668 (61,078 to -1,295,165^a^)	0.407 (1 to 0)
>6	0.69 (0.50 to 0.94)	215,056 (120,091 to 596,224)	0.04 (0.752 to 0)
Histology			
Serous	0.69 (0.52 to 0.93)	215,056 (127,497 to 564,006)	0.04 (0.668 to 0)
Non-serous	1.11 (0.15 to 7.91)	4548,227 (40,928 to -18,244^a^)	0 (1 to 0)
Region			
North America	0.69 (0.38 to 1.27)	231,616 (90,032 to -1,199,510^a^)	0.04 (0.985 to 0)
Europe	0.67 (0.46 to 0.98)	217,248 (114,837 to 820,105)	0.062 (0.879 to 0)
South Korea, Australia,and Latin America	0.76 (0.39 to 1.48)	293,169 (92,855 to -491,034^a^)	0.002 (0.976 to 0)

a The placebo group demonstrated greater efficacy while being less expensive, resulting in a negative ICER

*RnP* Relacorilant plus nab-paclitaxel, *nP* Nab-paclitaxel, *WTP* willingness-to-pay, *OS* Overall survival, *HR* hazard ratio, *CI* Confidence interval, *ICER* Incremental cost-efectiveness ratio, *QALY* Quality-adjusted life-year, *ECOG* Eastern Cooperative Oncology Group, *PARP* Poly (ADP-ribose) polymerase, *BRCA* Breast cancer gene

## Discussion

PROC remains a major clinical challenge in the U.S., characterized by high mortality and poor prognosis among gynecological malignancies. In alignment with key objectives of the Cancer Moonshot Initiative, which aims to improve quality of life for patients with advanced cancer and optimize healthcare resource allocation, the American Cancer Society and academic institutions have increasingly promoted the development of targeted and combination therapies for PROC. Despite recent advances in diagnostics and treatment, the 5-year survival rate for PROC patients remains under 30%, while the high cost of novel therapies continues to impose a substantial economic burden on the U.S. healthcare system, insurers, and patients [22, 37]. A global study estimates that cancer-related macroeconomic losses will reach $25.2 trillion from 2020 to 2050, with the U.S. accounting for a significant share. As the deadliest gynecologic malignancy, ovarian cancer contributes substantially to rising healthcare expenditures [38]. In this context, identifying treatment strategies that balance clinical benefit and economic sustainability is essential to mitigate both the clinical and financial burden of PROC.

Prioritizing cost-effective treatments and enhancing their clinical accessibility is essential for balancing therapeutic efficacy with healthcare system sustainability. As a first-in-class combination therapy inhibiting the GR pathway, the RnP regimen significantly improved PFS and OS in patients with PROC compared to conventional nP monotherapy in the Phase III ROSELLA trial, with no significant increase in safety risks [7]. As a novel option for this refractory disease, the pricing of RnP upon launch is bound to draw close attention from U.S. payers, clinicians, and patients. In the context of a global shift from efficacy-focused to value-based drug pricing [39], this study evaluates the cost-effectiveness of RnP to support payer negotiations, clinical decision-making, and insurance policy optimization, thereby improving access to high-value innovative therapies.

The base-case analysis revealed that the ICER of the RnP regimen versus the nP regimen for the treatment of PROC was calculated at $161,753/QALY, which exceeds the widely accepted WTP threshold of $150,000/QALY from the U.S. payer’s perspective. This indicates the RnP regimen is not cost-effective under current price assumptions. One-way sensitivity analysis confirmed that the cost-effectiveness of the RnP regimen is highly sensitive to the price of relacorilant, utility value in PFS and PD, while the impact of other parameters is relatively limited. The finding was further corroborated by PSA, which demonstrated robust and consistent results across simulations. The result of scenario analysis provided a definitive, evidence-based pricing benchmark: a reduction to $2.262/mg. At this price, which is approximately 92.2% of the current estimated cost, the regimen has a 51.73% probability of being cost-effective at the predefined WTP threshold. This provides explicit quantitative evidence for subsequent pricing negotiations in the U.S. market.

Subgroup analysis further identified populations that derived the greatest benefit, including patients aged > 65 years, those with a platinum-free interval ≤ 6 months, or a taxane-free interval ≤ 6 months. These populations generally presented a lower HR, indicating a lower risk of disease progression and a relatively favorable prognosis, in whom the RnP regimen provided superior clinical and economic value. Mechanistically, as a selective GR activation, relacorilant reverses glucocorticoid-mediated chemoresistance and enhances the cytotoxicity of nab-paclitaxel [8–10]. This synergistic effect is more prominent in patients with a lower HR, leading to improved PFS and quality of life, thus translating to greater QALY gains relative to incremental treatment costs, whereas the survival improvement in higher-HR subgroups is insufficient to offset relacorilant’s additional economic burden, resulting in inferior cost-effectiveness. Therefore, U.S. commercial insurance companies and federal healthcare programs (Medicare/Medicaid) could optimize reimbursement strategies based on these findings. In turn, clinicians should integrate subgroup results with patient-specific factors to maximize therapeutic efficacy and resource efficiency, enabling innovative therapies to precisely benefit eligible populations.

Previous cost-effectiveness analyses have been conducted for similar therapeutic regimens [40]. Notably, Zhou et al. evaluated the economic value of relacorilant‑containing regimens using data from a Phase II clinical trial [25]. In the present study, the ICER of the RnP regimen versus the nP regimen was estimated at $161,753/QALY, which differs notably from the ICER of $32,182.70/QALY reported by Zhou et al. The key reasons for this discrepancy may be as follows: First, in contrast to the small‑sample Phase II data used by Zhou et al., our study was based on large‑sample Phase III clinical evidence, which more accurately reflects real‑world clinical benefit [7]. Second, PROC is a refractory malignancy associated with a poor prognosis.The lifetime time horizon adopted by Zhou et al. for survival extrapolation may have overestimated long‑term benefits, whereas our 5‑year time horizon aligns with guidelines for oncology pharmacoeconomics and better reflects the natural disease course of this population [15]. Third, the studies used different assumptions for drug cost estimation. Zhou et al. estimated costs based on a daily dose conversion between mifepristone and relacorilant, whereas our analysis used clinically validated cycle‑based dose equivalence ratios derived from actual clinical administration schedules, yielding estimates that may be more consistent with real‑world practice [7, 26]. Furthermore, our study also offers the following advantages: We established an evidence-based pricing benchmark (relacorilant at $2.262/mg), which can provide a reference for drug pricing in the United States, and performed subgroup analyses to pinpoint populations (e.g., aged > 65 years) with enhanced economic value, providing useful insights for clinical practice and healthcare resource allocation.

Several limitations of this economic evaluation should be considered when interpreting the findings. First, reliance on clinical inputs from the ROSELLA trial may limit the generalizability of the results to real-world populations, particularly those with significant comorbidities or a higher number of prior treatment lines than allowed by the trial protocol. Real-world effectiveness may differ from trial efficacy, which could alter long-term cost-effectiveness estimates. Second, the study population was derived from the global ROSELLA trial enrolling patients across 14 countries in three regions (North America, Europe, and South Korea/Australia/Latin America), whereas our cost-effectiveness analysis adopted a U.S. payer perspective. This may introduce population heterogeneity. Although regional subgroup analyses confirmed that the nP regimen was not cost-effective across all three regions, supporting the robustness of the base-case results, dedicated clinical data for the U.S. population would further enhance the reliability of our findings. Third, survival data were extrapolated beyond the trial follow-up period using a parametric model selected based on statistical fitting criteria and visual inspection, despite this being a standard practice. Fourth, owing to the lack of detailed time-to-event data for SAEs from the ROSELLA trial, the model assumed that all grade ≥ 3 SAEs with an incidence > 3% included in our study occurred in the first treatment cycle. This simplification is widely accepted in oncology cost-effectiveness analyses, as the first treatment cycle is generally associated with a higher risk of treatment-related AEs [41–43]. Although this assumption may potentially underestimate the economic impact of AE management costs, one-way sensitivity analysis demonstrated that AE-related costs had only a modest impact on model outcomes. Furthermore, some model parameters in Table 1 were initially derived from single data sources. These factors may introduce minor bias in cost and QALY estimates. However, PSA confirmed that these parameters were not key drivers of the results. Finally, as relacorilant has not yet been marketed, its price was estimated based on the equivalent efficacy ratio of mifepristone. A launch price higher than the estimate would further reduce cost-effectiveness. Nevertheless, multi-stakeholder collaboration to develop rational pricing strategies, optimize medical insurance reimbursement policies, and refine individualized clinical decision-making holds promise for enabling more patients to benefit from this innovative therapy while safeguarding the sustainability of the U.S. healthcare resources. In summary, future research should conduct prospective economic evaluations in U.S. real-world settings to further validate the conclusions of this study, thereby providing more robust evidence to support the clinical implementation of the regimen and the formulation of related policies.

## Conclusion

The RnP regimen provides significant survival benefits in PROC. However, based on the current estimated pricing, its cost-effectiveness ratio exceeds the WTP threshold of $150,000/QALY. Analyses suggest that if the price of relacorilant is reduced to $2.262/mg, the regimen would approach cost-effectiveness. It is recommended that payers use this price as a reference for negotiation, clinicians select appropriate patient populations considering cost-effectiveness, and pharmaceutical companies explore value-based pricing strategies.

## Supplementary Information

Supplementary Material 1.

## Data Availability

All authors had full access to all of the data in this study and take complete responsibility for the integrity of the data and accuracy of the data analysis. The datasets generated and/or analyzed during the current study are available from the corresponding author upon reasonable request.
