# Supplementary material for: Cost-effectiveness analysis of nab-paclitaxel with or without relacorilant for platinum-resistant ovarian cancer
Source: J Ovarian Res. 2026 Apr 23;19:203. doi: 10.1186/s13048-026-02109-7 (PMC13237943; doi:10.1186/s13048-026-02109-7)
Supplement: Supplementary file 1 — Supplementary Material 1. [file 13048_2026_2109_MOESM1_ESM.docx]

**
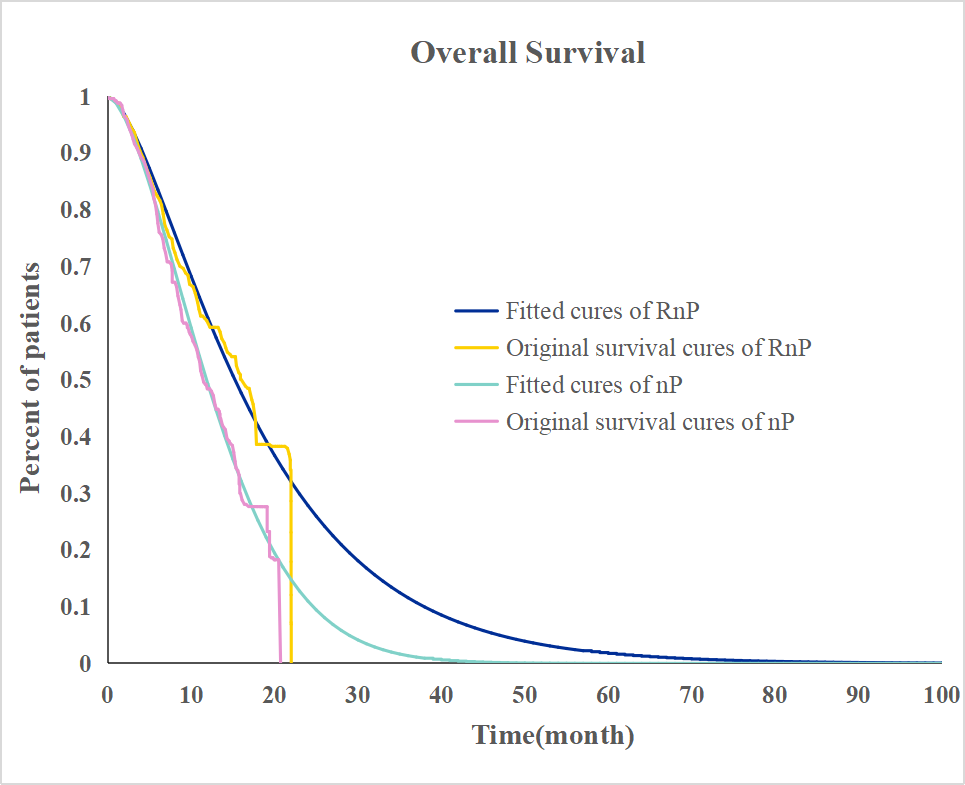
a**

**
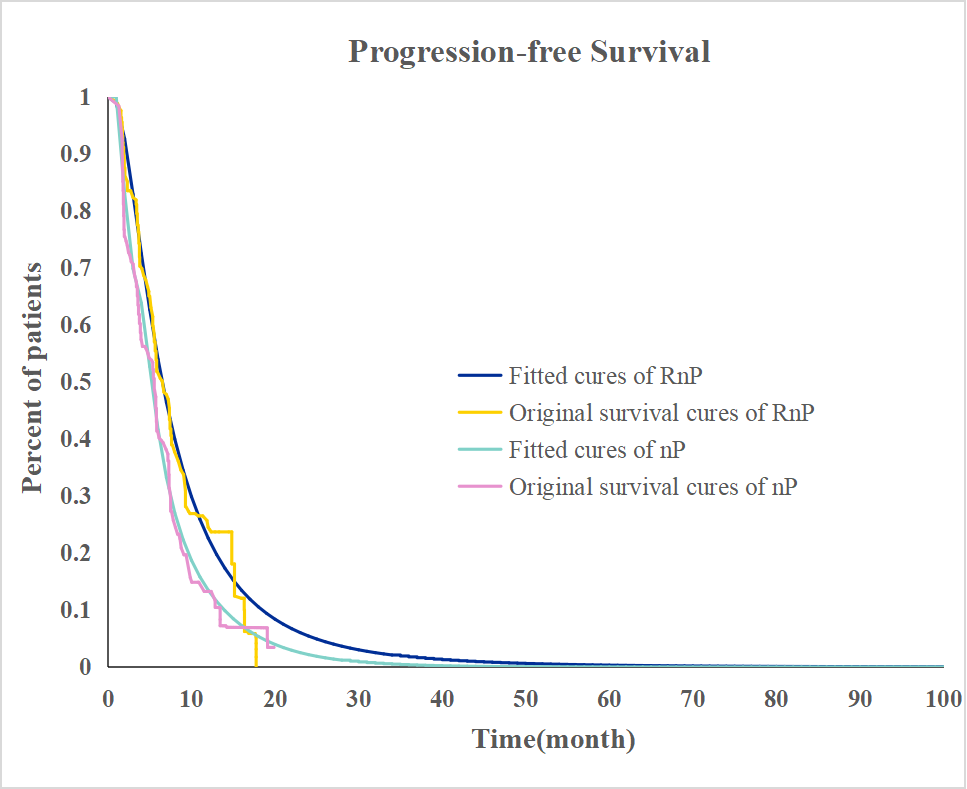
b**

**Supplementary Figure S1** The fitted and original Kaplan-Meier curves

(a: Overall Survival; b: Progression-free Survival)

RnP: Relacorilant plus nab-paclitaxel; nP: Nab-paclitaxel
